# Functional connectivity between white matter and gray matter based on fMRI for Alzheimer's disease classification

**DOI:** 10.1002/brb3.1407

**Published:** 2019-09-11

**Authors:** Jie Zhao, Xuetong Ding, Yuhang Du, Xuehu Wang, Guozun Men

**Affiliations:** ^1^ College of Electronic and Information Engineering Hebei University Baoding China; ^2^ Research Center of Machine Vision Engineering & Technology of Hebei Province Baoding China; ^3^ Key Laboratory of Digital Medical Engineering of Hebei Province Baoding China; ^4^ School of Economics Hebei University Baoding China

**Keywords:** Alzheimer's disease, dynamic functional connectivity, resting state fMRI, support vector machine, white matter

## Abstract

**Introduction:**

Alzheimer's disease (AD) is a chronic neurodegenerative disease that generally starts slowly and leads to deterioration over time. Finding biomarkers more effective to predict AD transition is important for clinical medicine. And current research indicated that the lesion regions occur in both gray matter (GM) and white matter (WM).

**Methods:**

This paper extracted BOLD time series from WM and GM, combined WM and GM together for analysis, constructed functional connectivity (FC) of static (sWGFC) and dynamic (dWGFC) between WM and GM, as well as static (sGFC) and dynamic (dGFC) FC within GM in order to evaluate the methods and areas most useful as feature sets for distinguishing NC from AD. These features will be evaluated using support vector machine (SVM) classifiers.

**Results:**

The FC constructed by WM BOLD time series based on fMRI showed widely differences between the AD group and NC group. In terms of the results of the classification, the performance of feature subsets selected from sWGFC was better than sGFC, and the performance of feature subsets selected from dWGFC was better than dGFC. Overall, the feature subsets selected from dWGFC was the best.

**Conclusion:**

These results indicated that there is a wide range of disconnection between WM and GM in AD, and association between WM and GM based on fMRI only is an effective strategy, and the FC between WM and GM could be a potential biomarker in the process of cognitive impairment and AD.

## Graphical abstract

White matter (WM) and gray matter (GM) was combined together for analysis based on fmri only, and the dynamic functional connectivity between WM and GM (dWGFC) was constructed. The feature subset selected from dWGFC had the best classification performance and generalization performance, which indicated the FC between WM and GM could be a potential biomarker in the process of cognitive impairment and AD.

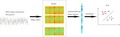

## 1 INTRODUCTION

Alzheimer's disease (AD) is considered to be the most common type of dementia in the elderly population. Its main features are brain atrophy, loss of neurons and synapses, plaque deposition, and neurofibrillary tangles containing tau protein. AD is usually divided into the following three periods: early, middle, and late. The deterioration related to this disease in patients is accompanied by changes in the white matter (WM) and gray matter (GM) of the brain morphology.

DTI technology is currently recognized as a noninvasive method for displaying WM fiber bundles, which can quantitatively analyze the diffusion properties of water molecules in WM to reflect the structural trend of nerve fibers, and plays an important role in detecting and diagnosing AD (Lo et al., 2010; Nowrangi et al., 2013). The characteristics extracted from DTI have also been studied for the classification of mild cognitive impairment (MCI) and AD,and have achieved some results (Jung, Lee, Kim, & Mun, 2015; Wee et al., 2012). In addition, fMRI has also been used as an imaging technique based on blood oxygen level‐dependent contrast (BOLD) signals for detecting human cortical nerve activity (Heeger & Ress, 2002). The brain function network based on fMRI mainly studied the connection between GM regions in different states. The analysis of functional connectivity (FC) between GM in AD patients has a positive impact on revealing the pathological process of AD (Liu, Yu, Zhang, Liu, & Duan, 2014; Scherr et al., 2018).

In recent years, increasing literature has reported WM activities detected by fMRI. For example, activation in the posterior limb of the internal capsule was observed in a magnetic field of 4T (Mazerolle et al., 2013); on the other hand, Tettamanti et al. used specialized task paradigms to detect enhancement of BOLD signals in WM (Tettamanti et al., 2002). Ding et al. observed similar temporal and spectral profiles between GM and WM at rest (Ding et al., 2013) and observed that their relative low‐frequency (0.01–0.08 Hz) signal powers were comparable (Ding et al., 2016). Then, Chen et al. (2017) constructed a functional correlation tensor of WM based on the BOLD signal and used it for the classification of MCI to obtain better classification performance. Furthermore, Zhang et al proposed a noise‐robust functional correlation tensor based on BOLD signals in the white matter and demonstrated its utility in AD diagnosis (Zhang et al., 2017). Ding et al. (2018) compared the changes in BOLD signals between WM under a resting state and WM under a visual stimulation task state, and the synchronous activity and correlation between WM and GM were studied, which provided strong evidence that BOLD signals in WM can reflect neural activity under different states and demonstrated that the fluctuation in the BOLD signals in WM is significantly correlated with specific GM regions. Huang et al. (2018) detected WM activations under visual stimulation by analyzing the spatial distributions of BOLD signal frequency spectra, confirming that BOLD signal variations in WM are modulated by neural activity.

**Figure 1:**
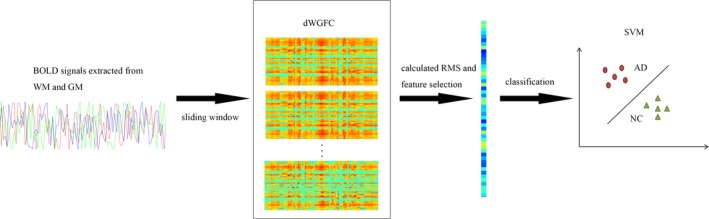
Framework of the proposed method

Inspired by these results, the current paper examined static and dynamic FC within GM and between WM and GM in order to evaluate the methods and areas most useful as feature sets for distinguishing NC from AD. These features will be evaluated using support vector machine (SVM) classifiers. The flowchart of the proposed method is shown in Figure 1.

## 2 METHODS

### 2.1 Materials and preprocessing of rs‐fMRI data

In this study, the data used were selected from the publicly available Alzheimer's Disease Neuroimaging Initiative (ADNI) database (http://adni.loni.usc.edu). Forty‐five AD patients were selected as the AD group, and 45 age‐ and gender‐matched normal cognitive subjects were selected as the NC group. The specific information is shown in Table 1.

**Table 1:** Information of the subjects included in this study

Group	Number	Gender (M/F)	Age	MMSE
AD	45	22/23	72.6 ± 7.1	21.24 ± 3.44
NC	45	20/25	74.3 ± 8.4	28.45 ± 1.82

The fMRI images of each subject were obtained with 3.0 T scanners. Images were acquired using an echo planar imaging (EPI) sequence, it's repetition time is 3 s, echo time is 30 ms, flip angle is 80, slices = 48, and field of view is 212 mm RL, 198.75 mm AP, and 159 mm FH. The voxel size was 3.13 × 3.13 × 3.13 mm^3^, and the SPM12 software package (http://www.fil.ion.ucl.ac.uk/spm/software) was used to preprocess the rs‐fMRI data. First, the images were corrected for slice timing and head motion, and subjects with substantial head motion (larger than 2 mm or 2°) were removed from the analysis. Then, T1‐weighed images were segmented into GM and WM, and then, these images were registered to the corrected BOLD image. Next, the fMRI images were normalized into Montreal Neurological Institute (MNI) space, and the images were then spatially smoothed with a Gaussian kernel with full width at half maximum of 6 × 6 × 6 mm^3^. And a bandpass filter was used to reduce the effects of low‐frequency drift and high‐frequency physiological noise, a signal of 0.01–0.1 Hz was obtained.

### 2.2 Construction of functional connectivity

Eighty‐two ROIs in GM were selected by the Brodmann atlas, and 48 ROIs in WM were selected using the JHU ICBM‐DTI‐81 atlas. By calculating the average BOLD time series of all voxels in each ROI, the regional average BOLD signal was obtained, which reflects the regional neural activity at the corresponding time.

Since rs‐fMRI reflects an unstable spontaneous cognitive activity, static FC loses some information, which may underestimate the complex and dynamic interaction patterns between different brain regions, and dynamic FC changes over time reflecting additional and rich information about brain activities. Therefore, a sliding time window strategy, a popular approach (Chen et al., 2016, 2017; Leonardi et al., 2013; Wee, Yang, Yap, & Shen, 2016), was used to construct dynamic FC, while static FC was also obtained as a comparison. Specifically, for each subject, the Pearson correlation coefficient, denoted as *r*, of each ROI pair between GM was calculated directly to construct a static FC of GM (sGFC). And static FC between GM and WM (sWGFC) was obtained by calculating each ROI pair's *r* between WM and GM. Then, a sliding time window strategy was applied, and a complete time series was divided into several subsequent series by overlapping windows. We allowed the window length to be 30 and the step size to be 1. Then, the correlation coefficient of each subsequence was calculated and denoted as ${\text{FC}}_{\mathrm{ij}}=\left[C_{\mathrm{ij}}^{1}\ldots C_{\mathrm{ij}}^{k}\ldots C_{\mathrm{ij}}^{K}\right]$, where *i* and *j* represent the *i*‐th and *j*‐th ROI, and *k* represents the *k*‐th subsequence. A complete time series is divided into *K* subsequences. Next, the RMS feature of FC was calculated by the following formula:

$$\text{RMS}\left({\text{dFC}}_{\mathrm{ij}}\right)=\sqrt{\frac{\sum_{k=1}^{K}{\left(C_{\mathrm{ij}}^{k}\right)}^{2}}{K}}$$

The RMS value is also known as the quadratic mean in mathematics and reflects the fluctuation level of the signals (Chen et al., 2017; Dey & Tech, 2014). As a result, the dGFC and dWGFC of each subject were obtained.

### 2.3 Feature selection

**Figure 2:**
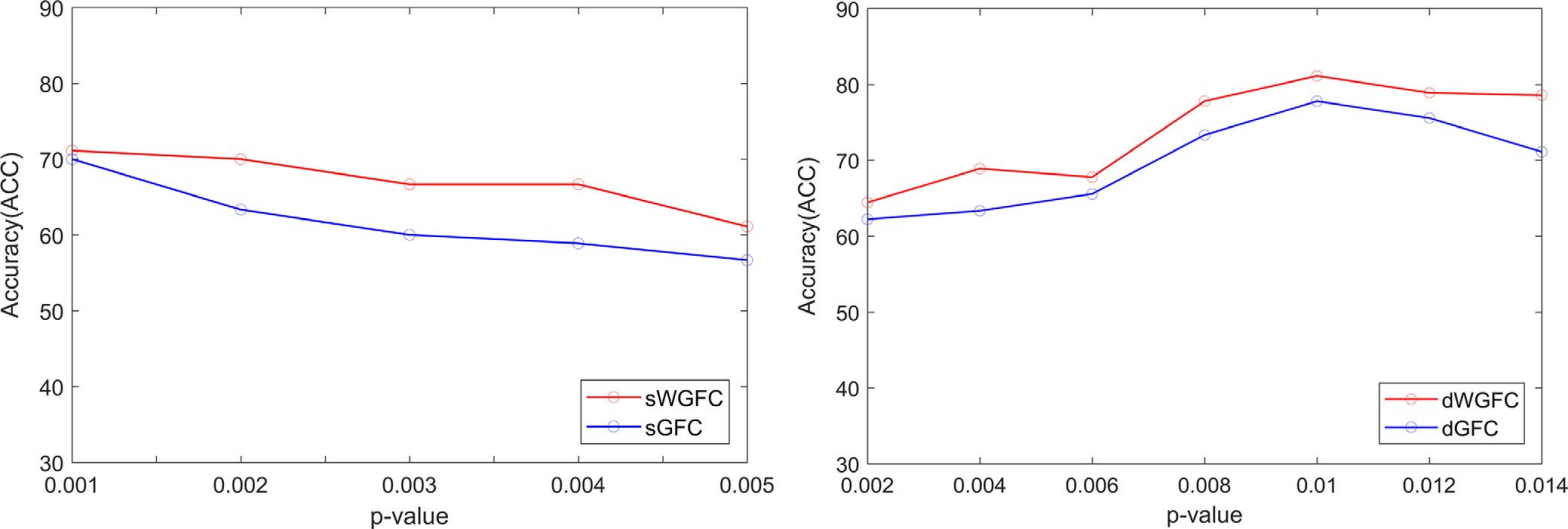
Different *p*‐value and corresponding accuracy

In the next step, Fisher's *r*‐to‐*z* transformation, which is expressed as $z=\left[\ln\left(1+r\right)-\ln\left(1-r\right)\right]/2$, was applied to all FC matrices, including static and dynamic, to improve the normality of the correlation coefficients. Then, a two‐sample *t* test was used to select ROI pairs with significant differences to be used as the feature subset for the classification test. For sFC and dFC, we tested different *p*‐value and corresponding accuracy, selected the feature subset with the highest accuracy. As shown in Figure 2, so the features selected from sGFC and sWGFC were chosen when *p* ≤ .001, and those from dGFC and dWGFC were chosen when *p* ≤ .01.

### 2.4 Classifier learning

In this experiment, the main purpose was to compare feature subsets that contribute more to distinguishing AD from NC, rather than focusing on better classification models. Therefore, the classifier was not optimized, and the SVM classification model was selected and LIBSVM library was performed to implement SVM classification. Studies (Yang, 2004) have shown that SVM classifiers have advantages for bioinformatics research. The SVM classifier in this paper selected the linear kernel function. Due to the limited samples, a leave‐one‐out (LOO) cross‐validation was applied to benchmark the different performance of different feature subsets.

## 3 RESULTS

### 3.1 Comparison of sWGFC

**Figure 3:**
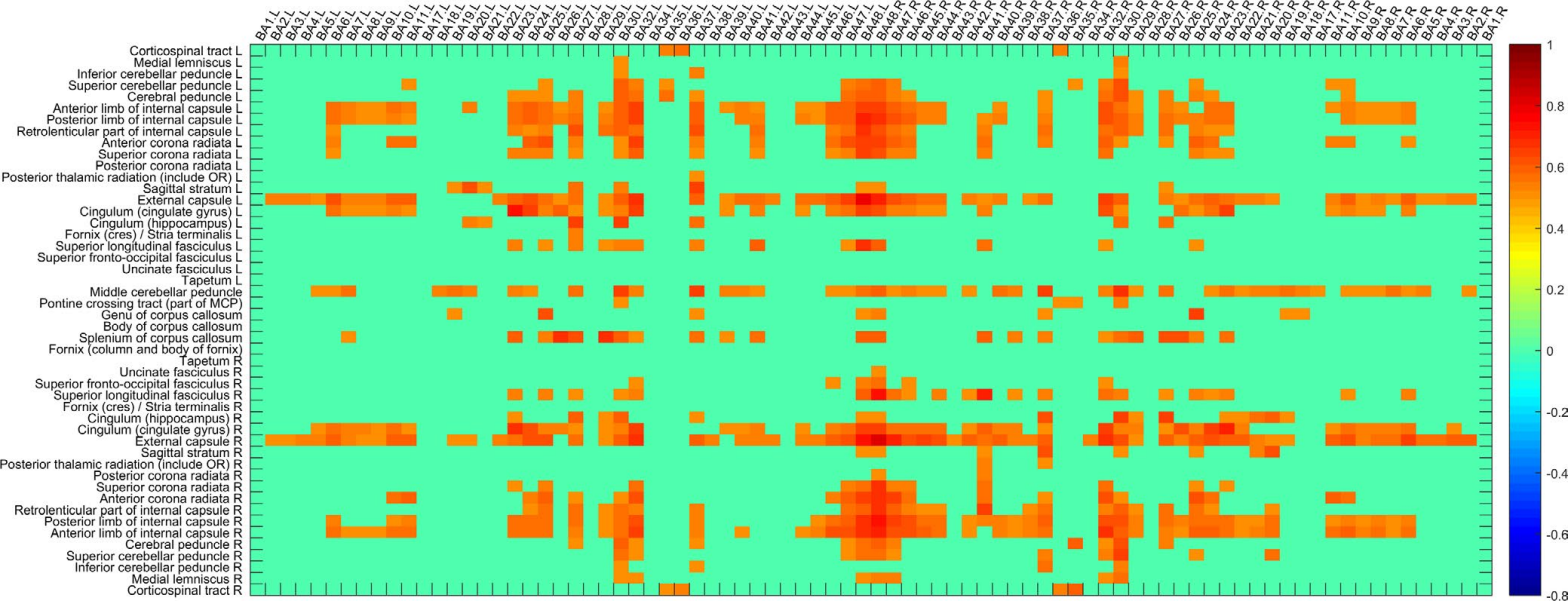
sWGFC of the NC group (|*r*| > 0.5)

**Figure 4:**
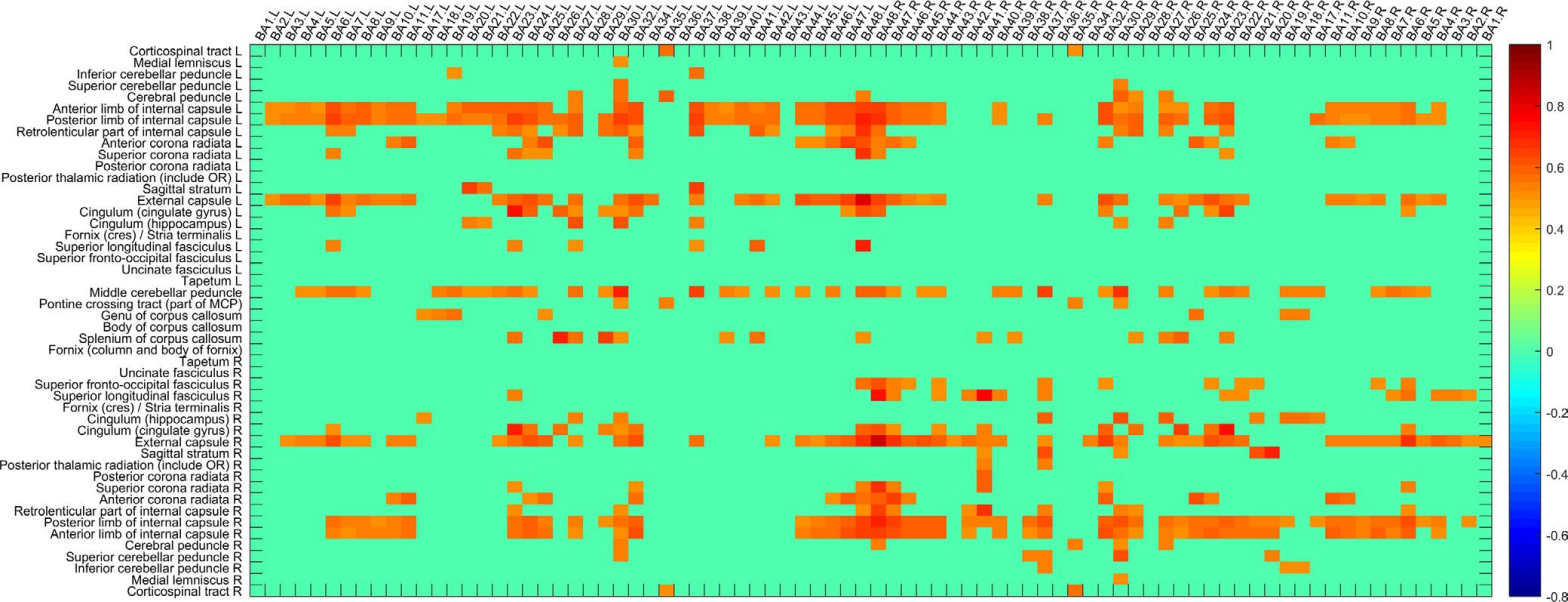
sWGFC of the AD group (|*r*| > 0.5)

Without a sliding time window strategy, the sWGFC of the NC group and AD group was calculated, as shown in Figures 3 and 4, as in sWFC, where *r* is greater than the set threshold was displayed. Obviously, the sWGFC of the AD group has more loss of connection than that of the NC group.

### 3.2 Comparison of dWGFC

**Figure 5:**
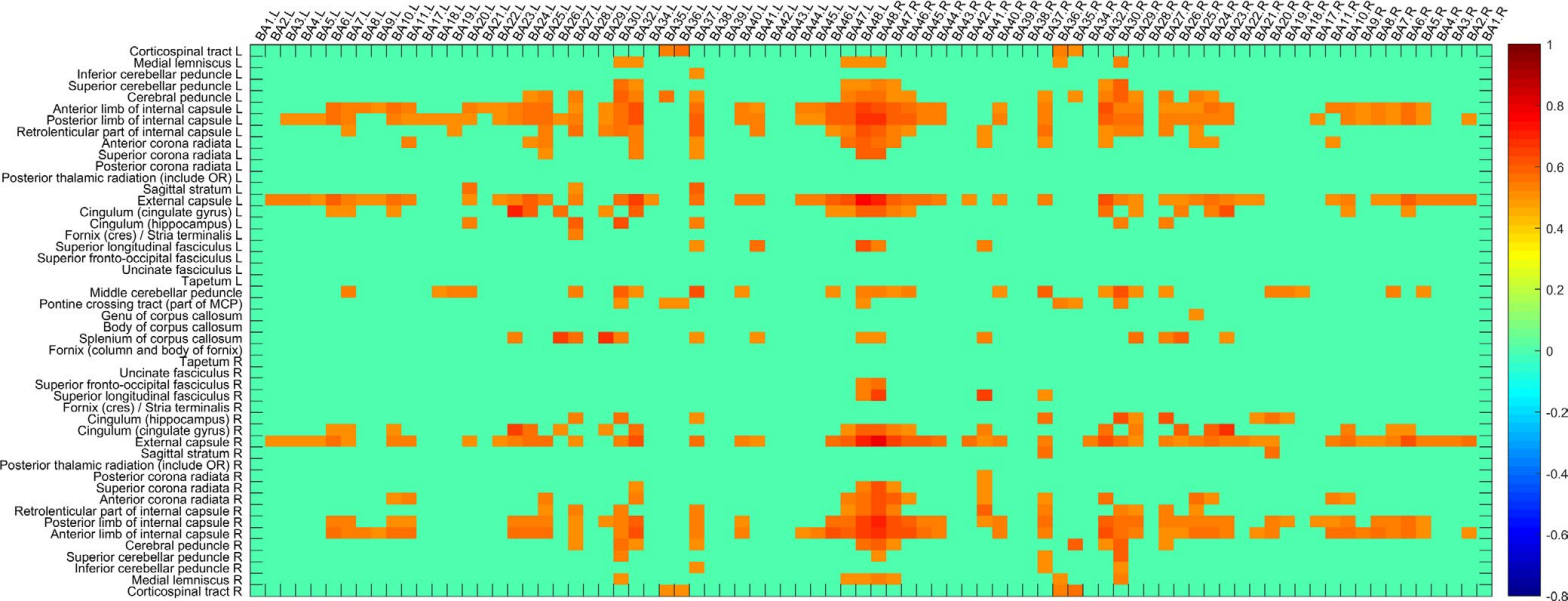
dWGFC of the NC group (|RMS| > 0.5)

**Figure 6:**
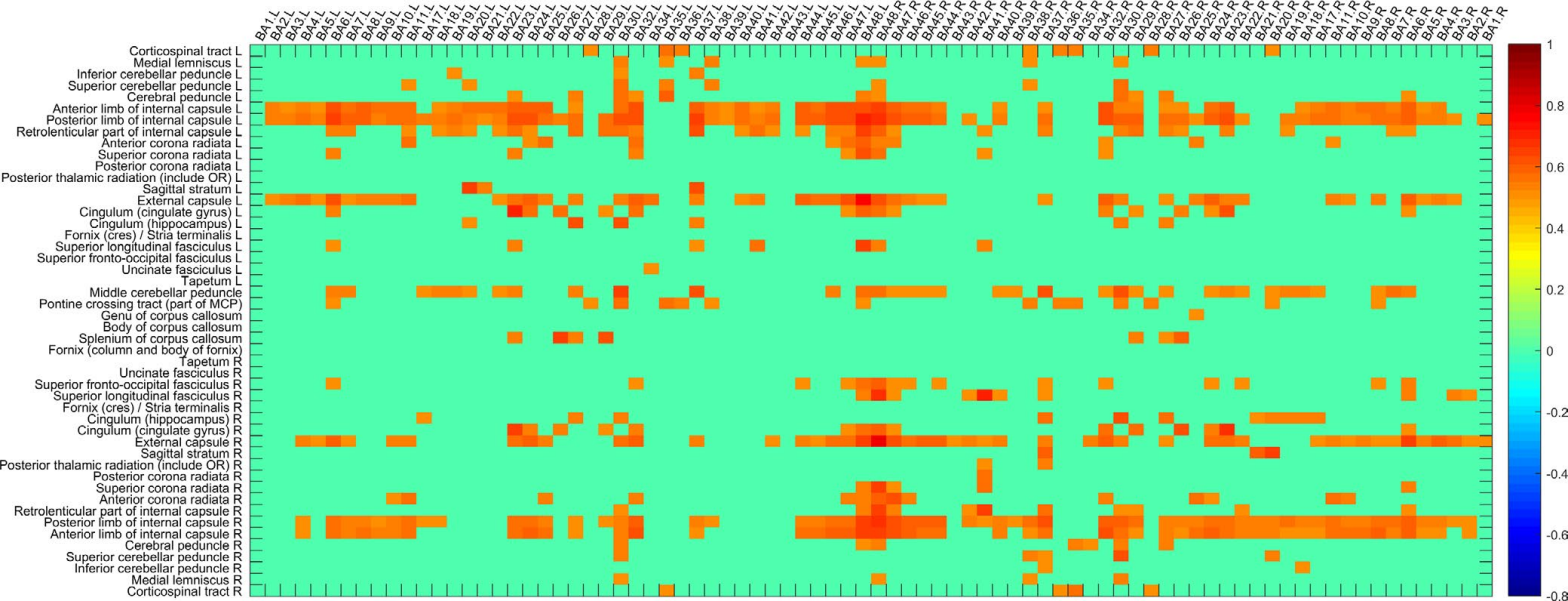
dWGFC of the AD group (|RMS| > 0.5)

A sliding time window strategy was applied, the mean RMS value of the AD group and NC group was obtained, as shown in Figures 5 and 6, where RMS is greater than the set threshold was displayed. There are many areas that have differences in the two groups, and these features will form feature subsets for classification.

### 3.3 Classification performance of different feature subsets

**Figure 7:**
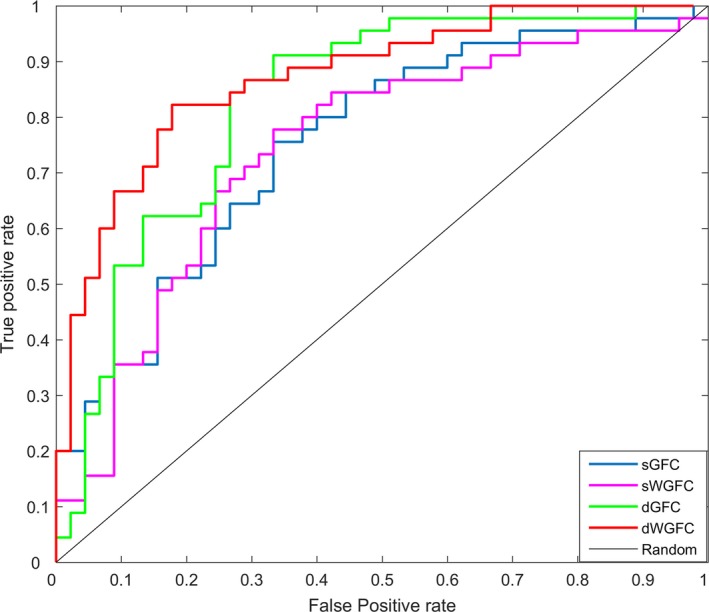
ROC curves

Four feature subsets were selected from sGFC, sWGFC, dGFC, and dWGFC. The features selected from sGFC and sWGFC were chosen when the *p*‐value was <.001, and those from dGFC and dWGFC were chosen when the *p*‐value was <.01. The features were tested with the SVM classifier, and their performance is shown in Table 2. We evaluated the performance of different subsets from the following five indices: accuracy (ACC), sensitivity (SEN), specificity (SPE), area under the receiver operating characteristic curve (AUC), and *F*‐score. And the definitions of ACC, SEN, SPE, and *F*‐score are given as follows:

$$\text{ACC}=\frac{\text{TP}+\text{TN}}{\text{TP}+\text{TN}+\text{FP}+\text{FN}}$$

$$\text{SEN}=\frac{\text{TP}}{\text{TP}+\text{FN}}$$

$$\text{SPE}=\frac{\text{TN}}{\text{TN}+\text{FP}}$$

$$F-\text{score}=2\times \frac{\text{precision}\times \text{recall}}{\text{precision}+\text{recall}}$$

where $\text{precision}=\frac{\text{TP}}{\text{TP}+\text{FP}}$, and $\text{recall}=\frac{\text{TP}}{\text{TP}+\text{FN}}$. The feature subsets from dWGFC had higher accuracy and better generalization performance. Figure 7 plots the receiver operating characteristic (ROC) curves for different subsets. In addition, the top 15 features selected from dWGFC are displayed, as shown in Table 3.

**Table 2:** Performance of different methods in classification

Method	ACC (%)	SEN (%)	SPE (%)	AUC	*F*‐score
sGFC	70	62.22	77.78	0.7309	67.46
sWGFC	71.11	62.22	80	0.7427	68.30
dGFC	77.78	64.44	91.11	0.8059	74.36
dWGFC	81.11	84.44	77.78	0.8499	81.72

**Table 3:** Top 15 ROI pairs selected from dWGFC during classification (*p*‐value was corrected used FDR [*q* = 0.05])

WM	GM	*p*‐value
Posterior limb of internal capsule **L**	Includes frontal eye fields **L**	.0016
Sagittal stratum **L**	Piriform cortex **L**	.0066
Uncinate fasciculus **L**	Retrosplenial cingulate cortex **R**	.0071
Genu of corpus callosum	Anterior prefrontal cortex (most rostral part of the superior and middle frontal gyri) **L**	.0069
Genu of corpus callosum	Part of the perirhinal cortex (in the rhinal sulcus) **R**	.0058
Genu of corpus callosum	Anterior prefrontal cortex (most rostral part of the superior and middle frontal gyri) **R**	.004
Body of corpus callosum	Fusiform gyrus **R**	.0035
Superior fronto‐occipital fasciculus **R**	Superior temporal gyrus, of which the caudal part is usually considered to contain the Wernicke's area **R**	.0046
Superior longitudinal fasciculus **R**	Premotor cortex and supplementary motor cortex (secondary motor cortex) (supplementary motor area) **R**	.0032
Superior longitudinal fasciculus **R**	Primary motor cortex **R**	.0041
Superior longitudinal fasciculus **R**	Primary somatosensory cortex2 **R**	.0036
Superior longitudinal fasciculus **R**	Primary somatosensory cortex 1 (frequently referred to as Areas 3, 1, and 2 by convention) **R**	.0066
Fornix (cres)/ Stria terminalis **R**	Dorsal entorhinal cortex (on the parahippocampal gyrus) **L**	.0065
Sagittal stratum **R**	Middle temporal gyrus **R**	.00092
Sagittal stratum **R**	Inferior temporal gyrus **R**	.0027

## 4 DISCUSSION

### 4.1 Comparison of classifier performance

As shown in Table 2, the SVM classifier used a linear kernel function, and LOO cross‐validation was applied. Feature subsets selected from dWGFC achieved a better balance between sensitivity and specificity, and its ACC was 81.11%, which is higher than that of the other three methods. Sensitivity reflected the ability to confirm AD patients, while specificity reflected the ability to confirm a normal control. A better balance between sensitivity and specificity reflected a better generalization ability of the feature subsets, which means that the association between the WM and GM signals based on fMRI has positive significance in distinguishing AD from NC.

### 4.2 Analysis of physiological significance

Table 3 shows that the areas involved in the WM included: the posterior limb of internal capsule, the sagittal stratum, the uncinate fasciculus, the genu of corpus callosum, the body of corpus callosum, the superior fronto‐occipital fasciculus, the superior longitudinal fasciculus, and the fornix. These abnormal phenomena are consistent with those reported in previous studies based on other technology (Fletcher, Carmichael, Pasternak, Maier‐Hein, & DeCarli, 2014; Habes et al., 2018; Medina et al., 2006; Shu, Wang, Qi, Maier‐Hein, & DeCarli, 2011; Xie et al., 2006).

The superior longitudinal fasciculus, inferior longitudinal fasciculus, and fronto‐occipital fasciculus belong to long contact fibers, which are connected with the cortex of the ipsilateral hemisphere. and its abnormality leads to damage of the nerve circuit and affects cognitive function, as well as visual spatial processing, object recognition and memory (Catani, Jones, Donato, & Ffytche, 2003). It is generally believed that the structural integrity of the cingulum, fronto‐occipital fasciculus, superior longitudinal fasciculus, and uncinate fasciculus is closely related to AD (Perea et al., 2016). The corpus callosum and fornix belong to combined fibers, that connect the left and right hemisphere cortices. The complex connection of corpus callosum fibers makes it possible for the functional integration of bilateral cerebral hemispheres to ensure normal development of human emotions and various cognitive activities. As a result, damage to different parts of the corpus callosum may lead to different symptoms (Mielke et al., 2009). The fornix is an important part of the limbic system, which plays an important role in situational memory, emotional behavior and cognition (Nestor, Fryer, Smielewski, & Hodges, 2003). Damage to the fornix will lead to cognitive impairment and memory decline in patients. Posterior thalamic radiation and corona radiata belong to projection fibers, and posterior thalamic radiation passes through the posterior limbs of the internal capsule. Damage to the posterior limbs of the internal capsule is closely related to motor and sensory dysfunction in AD patients.

In addition, the GM ROIs involved included the piriform cortex, the cingulated cortex, the anterior prefrontal cortex, the perirhinal cortex, the fusiform gyrus, the temporal lobe, the parahippocampal gyrus, and the motor and somatosensory cortex. These areas are closely related to olfaction, emotion, learning, memory, and motor, and sensory functions (Chang et al., 2016; Choo et al., 2010; Daulatzai, 2015; Golob, Miranda, Johnson, & Starr, 2001; Grady, Furey, Pietrini, Horwitz, & Rapoport, 2001; Suvà et al., 1999; Wolk, Das, Mueller, Weiner, & Yushkevich, 2017).

White matter is the area where nerve fibers congregate in the brain and carrying the transmission of information. The loss of connection between different ROIs reflects different degrees of brain damage, leading to different clinical manifestations. The FC between WM and GM can reflect this disconnection more comprehensive, which suggests that we should pay more attention to these changes in the process of cognitive impairment and AD.

### 4.3 Limitations

It should be noted that this study has some limitations. On the one hand, using other statistics to construct dynamic features and representing signals from different perspectives may improve the results of the signal analysis. On the other hand, the number of samples analyzed is not enough, and expanding the scope of the study can lead to more general conclusions. In addition, convolutional neural networks and deep learning have greater capacity in medical image analysis and disease detection, the above two methods are the direction of our future work.

## 5 CONCLUSIONS

This paper studied the FC between WM and GM in the AD group and NC group based on fMRI data. A sliding time window strategy was applied to construct dGFC and dWGFC. Then, a two‐sample *t* test and SVM classifier were used to test feature subsets from four different methods, the association between WM and GM improved the classifier performance. And 15 ROI pairs (*p* < .01) in dWGFC were observed. These results indicated that there is a wide range of disconnection between WM and GM, and the WM time series obtained from fMRI is helpful to study AD, and the FC between WM and GM could be a potential biomarker to predict the development of AD as well as distinguish AD from others.

## CONFLICT OF INTEREST

The authors declare that they have no conflict of interest.

## Data Availability

The data that support the findings of this study are available from the corresponding author upon reasonable request.
